# Nurse‐Surgeons’ Experiences Working in the Australian Public Health System: A Qualitative Exploration

**DOI:** 10.1155/jonm/2341474

**Published:** 2026-01-16

**Authors:** Tenber Grota, Adam Burston, Vasiliki Betihavas, Elisabeth Jacob

**Affiliations:** ^1^ School of Nursing, Midwifery and Paramedicine, Australian Catholic University, Sydney, New South Wales, 2060, Australia, acu.edu.au; ^2^ Nursing Research and Practice Development Centre, The Prince Charles Hospital, Chermside, Queensland 4032, Australia, qld.gov.au; ^3^ School of Nursing & Midwifery, University of Notre Dame Australia, Sydney, New South Wales, 2010, Australia, nd.edu.au

**Keywords:** Australia, nurse-surgeon, perioperative nursing, qualitative research, surgery, thematic analysis

## Abstract

**Aim:**

To explore the experiences and perceptions of nurse‐surgeons practicing in the Australian public health system.

**Background:**

Nurse‐surgeons occupy a unique and innovative role within perioperative healthcare, yet their integration is often complex and underexplored in the existing literature.

**Sources of Evidence:**

This qualitative study adhered to the consolidated criteria for reporting qualitative research checklist of the EQUATOR Network, involving five semistructured interviews analysed inductively through a reflexive thematic analysis. Ethical approval was obtained prior to the study.

**Discussion:**

Participant interactions with supervisors were positive, while relationships with other nurses and physicians evolved from initial antagonism to mutual recognition of value. Facilitators for integration included awareness of the role, collaboration, a standardised national credentialing pathway and government involvement. The barriers consisted of geographical limitations, obstructive medical associations, financial challenges, tall poppy syndrome within nursing and a superiority complex among physicians.

**Conclusion:**

The study offers valuable insights into the nurse‐surgeons’ experiences and perceptions, highlighting essential barriers and facilitators to their integration in the healthcare system.

**Implications for Nursing Practice:**

Findings may guide healthcare institutions in fostering collaborative interprofessional interactions and implementing standardised credentialing pathways for nurse‐surgeons.

**Implications for Health Policy:**

The insights may inform nursing associations and government bodies in addressing systemic barriers and advocating for policy changes to enhance surgical delivery in Australia and globally.

## 1. Introduction

Global surgical disparities leave over five billion people without adequate access to essential surgeries [1]. Despite being a critical aspect of universal healthcare [2], surgery remains underrepresented on the global health agenda. Each year, millions undergo surgery to prevent disease progression or death [3], yet over 18 million die from conditions treatable by surgery [4]. This highlights the urgent need for equitable surgical care. Recognising surgery as a global health priority is essential, and nurse‐surgeons—nurses trained to perform surgeries independently [5, 6]—offer a promising solution to healthcare disparities through collaboration and specialised skills. Integrating nurse‐surgeons into healthcare could enhance access to surgical care and reshape the future of healthcare.

Nurse‐surgeons, first mentioned vaguely in the early 1500s [7], began independently performing surgeries in the 1950s to address surgical shortages in Africa [6]. Although efforts to standardise their role declined in the 1990s, nurse‐surgeon practice expanded globally into the 21^st^ century [8–10]. In 2009, they were recognised as qualified nonphysician surgeons in the United Kingdom [11] and now perform various surgeries, including appendicectomy, caesarean section, herniorrhaphy, hysterectomy, laparotomy, biopsy, carpal tunnel release, colonoscopy, cystoscopy, gastroscopy and hysteroscopy in Africa, Asia, Europe, Oceania and North America [5].

Nurse‐surgeons have improved surgical outcomes by increasing access, reducing errors and enhancing patient satisfaction, especially in underserved areas. In developing countries, the World Health Organization’s [12] task shifting strategy boosted their role, while in developed nations like Australia, they emerged to address surgical demand and workforce challenges, including an ageing medical workforce and uneven surgeon distribution [13, 14].

In Australia, the first documented nurse‐surgeons performed endoscopies in 2004 as part of a national bowel cancer initiative, achieving a 96.2% completion rate with 202 of 210 surgeries completed unassisted by physicians and without complications [15]. The study did not compare complication rates between nurse‐surgeon–performed and physician‐performed endoscopies. Subsequent Australian investigations of nurse‐surgeon–performed endoscopy [16–18] reported similarly positive outcomes. Regarding surgical access, a study of a nurse‐led flexible cystoscopy service demonstrated a 65% reduction in urological surgery waitlists [19]. Cusack et al. [17] planned to examine the impacts of nurse‐surgeons on waitlists and access, but the data remained incomplete. These five sources of evidence represent the only known Australian literature on nurse‐surgeons.

The introduction of nurse‐surgeons in Australia has expanded surgical capacity, reduced wait times and addressed workforce shortages, particularly in rural, remote and underserved areas [15–19]. A qualitative exploration is therefore timely, as it can uncover the perceptions, experiences, readiness and concerns of stakeholders from the point of view of practising nurse‐surgeons in Australia regarding the wider adoption and standardisation of this evolving role. Such insights are essential to guide policy, training and safe integration of nurse‐surgeons into the Australian healthcare system. This study is the first qualitative exploration of Australian nurse‐surgeons which followed Phase 1—a national quantitative study on their roles and training [20]. This study (Phase 2) focused on exploring further their experiences and perspectives.

## 2. Materials and Methods

The aim of the study was to explore the experiences and perceptions of nurse‐surgeons practicing in the Australian public health system.

### 2.1. Design

This study adhered to the consolidated criteria for reporting qualitative research checklist [21] from the EQUATOR Network (Supporting Information 1). Semistructured interviews were conducted one‐on‐one and remotely via Microsoft Teams due to the geographic dispersion of Australian nurse‐surgeons, making direct observation unfeasible. This virtual approach was both cost‐effective for a nationwide study and allowed flexibility in using open‐ended questions. The interview protocol, informed by Phase 1 findings [20] and the study’s explanatory sequential mixed methods design requirements, was developed by the first author (TG) with input from the coauthors (AB, VB and EJ).

### 2.2. Sample and Setting

Phase 2 focused on participants from Phase 2, utilising purposive sampling [22]. Recruitment occurred from April to June 2022, with all Phase 1 participants invited via an anonymous popup window on REDCap to opt in for interviews. Out of 28, 16 responded affirmatively, leading to six consents and five completed interviews (Table 1). To qualify, participants needed to be Australian Health Practitioner Regulation Agency–registered nurse practitioners or registered nurses performing surgeries independently during data collection and must have completed the Phase 1 survey. All surgical specialities were included, while those in purely assisting roles were excluded.

**Table 1:** Participant characteristics.

ID	Sex	State	Speciality	Surgery performed independently	Other role
1	Female	Victoria	Cardiothoracic	Open and endoscopic saphenous vein harvesting and radial artery harvesting	Surgical assistant
2	Female	Queensland	Gastroenterology	Colonoscopy	Clinic, quality and safety
3	Female	Queensland	Gastroenterology	Colonoscopy	Clinic
4	Female	Victoria	Urology	Flexible cystoscopy	Clinic
5	Female	Queensland	Cardiothoracic	Radial artery harvesting and saphenous vein harvesting	Surgical assistant

### 2.3. Data Collection

From November 2022 to December 2022, the first author (TG) conducted all the interviews, with only the participant and TG present during each session. An interview guide (Supporting Information 2), organised into seven sections, was developed after Phase 1 analysis, including demographics, introduction, questions, probing, closing, observation and field notes [23]. TG’s field notes and ideas from each interview partly guided the subsequent ones. All five interviews were audio‐recorded, with live transcription enabled via Microsoft Teams and saved in OneDrive. TG manually crosschecked and verified the Microsoft Teams–generated transcripts. The transcripts were not returned to participants for comments. Interview durations ranged from 36 to 46 min. No follow‐up interviews were required.

### 2.4. Data Analysis

**Figure 1:**
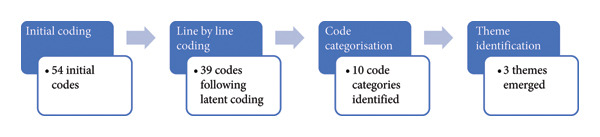
The coding tree.

The data were analysed inductively using a reflexive thematic analysis [24], with coding conducted in Microsoft Word. No automated coding software was used. Familiarisation with the data involved reviewing audio recordings and transcripts, while maintaining participant anonymity. Coding was iterative and collaborative, incorporating feedback throughout the process. In line with Braun and Clarke’s [25] recommendations, data saturation was not employed. Therefore, after completing five interviews from the six participants who had consented, further follow‐up with the one nonresponding participant was considered unnecessary. Initial coding yielded 54 semantic codes, which were refined to 39 through latent coding. Themes were developed through discussions that encouraged reflection on preconceptions about nurse‐surgeons in Australia. A report detailing the themes and direct quotes was produced, and a coding tree illustrating the process is shown in Figure 1. To validate the interview protocol, pilot testing was conducted, and the three coauthors (AB, VB and EJ)—who supervised the first author (TG)—provided methodological support to TG throughout the development of the protocol and the interview process. Reflexivity was maintained [26] through spontaneous notes, immediate documentation of reflections and the development of a reflexive statement (Supporting Information 3).

### 2.5. Ethical Considerations

Prior to recruitment, the study received approval from the Australian Catholic University’s Human Research Ethics Committee to be conducted from March 2022 to August 2023, with ethics register number 2022‐2426E. All participants were required to provide consent prior to the interview. None of the authors (TG, AB, VB and EJ) had any personal connections with the participants.

## 3. Results

Three main themes emerged from the research, highlighting the experiences and perceptions of nurse‐surgeons regarding their training, education, support and integration into the Australian health system: (1) nurse‐surgeon interactions, (2) facilitators for integration and (3) barriers to integration. Exemplary quotes supporting each theme and subtheme are presented in Table 2.

**Table 2:** Themes, subthemes and quotes.

Theme	Subtheme	Quotes (Participant ID)
Nurse–surgeon interactions	Interactions with healthcare professionals	‘It was hard for us as well because we wanted to, as experienced scrub scouts, we wanted to help out in that role too. So, we were kind of torn between learning cardiac and as a scrub scout, which we didn′t need to do.’ (Participant 1)
		‘It′s not something that we′re taught to do, and you know, giving a diagnosis cause nurses don′t diagnose. The first patient that I had found bowel cancer, I had to sit there and tell them that you′ve got cancer. So again, that′s not something that we′re trying to do but it was something that I had to learn. And another thing that I found quite difficult was that transition from the nurse to then being the person that′s managing the theatre, this is my theatre. This is how it′s going to be run and that includes, you know, giving direction to the anaesthetist.’ (Participant 2)
		‘There was definitely a head‐to‐head with the nurses. That was actually quite challenging dealing with the backlash from the nurses. When I first started, I think everyone was a little bit hesitant because they weren′t sure how it was working, like I was almost stepping on their toes in a way. They were nurses who had been there for 20 years or more than that, and so they were very experienced. And then I came in not knowing anything of the specialty but doing a more senior role. It was quite stressful and, you know, threatening for them.’ (Participant 1)
		‘It was quite challenging because obviously it′s a new role. It′s a pioneering role. Umm, there′s lots of pushback from both medical and nursing.’ (Participant 2)
		‘In the beginning they couldn′t get their heads around. They were, they didn′t understand it. They didn′t view us as lone accredited clinicians. We will walk into a flexi room. Ready to do a list and they′ll say oh, the nurses have called in sick so you′re gonna have to set up your own trolley. And I question, would you do that to a medical doctor? No, we just cancelled the list. And I said, well, that′s what you do here, we are the primary physician here. We have to focus on the clinical side of doing the flexi for the patient and they just don′t understand it.’ (Participant 4)
		‘And now the nurses are grateful for our communication.’ (Participant 1)
		‘I think they now see me as that role model within the nursing profession.’ (Participant 2)
		‘In the unit where I worked, a lot of the nurses were inspired and so a lot of them did more formal postgraduate training, a lot of them. Like I think half our unit went and did a postgraduate course in gastroenterology like that was just such a high number of nurses moving into postgraduate training and a few have gone on to do masters. Maybe not as nurse practitioners, but they value what that opportunity gives them.’ (Participant 3)
		‘But when we finally got in there, they were all in awe that we could do this. By the end, we kind of had won them over.’ (Participant 4)
		‘Surgeons who have us are appreciative of us and so encouraging of us the whole time.’ (Participant 1)
		‘When I started up the rectal bleed clinic, I did that under a colorectal surgeon’s tutorage. So, he had his clinic sitting alongside mine and he supported that clinic. If I had questions, he′d come in and review patients with me. So entirely collaborative. He was very supportive. I think my role was rather unique, though I was really supported in that environment.’ (Participant 3)
		‘I had a terrible complication, and it wasn′t because of the harvest. It was because the patient had already had an injury in their forearm that they hadn′t disclosed, that they had some neurological events after that. Umm, that took a long time to resolve and as a resolve of me operating on their forearm and it could have been any operation on being told they got a sympathetic reflex dystrophy, which is quite a catastrophic complication. And the patient had to have fasciotomies of their forearm and their hand, and it was all extremely traumatic. And I nearly walked away from me, you know, participating in that more invasive role, but I got excellent support from the surgeon.’ (Participant 5)
		‘So, the surgeons that I knew well were great and probably didn′t change over the course of me getting experience.’ (Participant 5)
		‘It was not a backlash, but just more of an irritation from the junior registrars.’ (Participant 1)
		‘They don′t understand what we do. They don′t understand our service. They don′t understand our capabilities and the fact that HMO, who has seen one done one, you know, teach one. What does it do? They aren′t as good as someone who has done, you know, we probably did 10,000 flexis and they can′t get their heads around that and there is somewhat a superiority complex in regard to that. At the moment we could do better than what some of the registrars and fellows are doing.’ (Participant 4)
		‘The doctor presented data and didn′t even acknowledge us. He absolutely took our figures and twisted them with wrong information yet was lauded as this great piece. A great poster and you know, won prize. And it′s like this is r– . This is inaccurate information.’ (Participant 4)
		‘There was a small population of medical surgical assistants who didn′t think I should be doing the role.’ (Participant 5)
		‘I think that over time they have accepted my capabilities, and I think that they probably respect it, to be perfectly honest. If that role can help the surgeon, then they′ll support it. But if they feel that the role is going up against their role well obviously there′s going to be pushback. And that′s I think with my role, it was more taking their job of doing exactly what they′re doing, not they′re doing the procedure and then they′re allowing you to close up or you know what I mean? Like I′m doing exactly what they′re doing. I think it it′s a lot of that fear factor of you know, the nurses are going to take over.’ (Participant 2)
		‘It′s not you, it′s the fact that it′s a nurse doing this advanced thing. And anytime I had that negativity, that′s what I would say. It′s not me. It′s not me they dislike, it′s what I′m doing.’ (Participant 3)
		‘I went door knocking because I wanted to move to this area and I′m supported fully by only two members of the gastroenterology team of about 10 gastroenterologists and the others really don′t want to have much to do with me, which is a little bit disappointing. But you know the surgeons appear to appreciate the work and I refer on to them. However, there′s not a lot of collaboration. I′m not invited to meetings, so that ongoing training that you need in your workplace is missing.’ (Participant 3)
		‘So if we have any issues from the registrars and fellows that is soon worked out by the consultant and the deputy head and the head of the unit.’ (Participant 4)
		‘I picked up a couple of surgeons who didn′t know me and I think that they were a bit reserved about having a nurse doing those very invasive procedures initially but gave me the leeway to actually do the job that I could do, and they′ve come to a point now where, you know, they will often say, oh, thanks very much for your help. I′m not sure I could have done that without you. I had a surgeon say that just a couple of months ago, we did a really complicated case and they′re very quick to ask for suggestions because I′ve worked with lots and lots and lots of cardiac surgeons. You know, if we get into a situation that′s particularly difficult, they will go oh what do you think I should do here? It′s like, well, I think you should do this, this and this. And sometimes they take your advice and sometimes they don′t. But they ask. So, yeah. So, I′m now at a position I′ve been doing it long enough that there′s not many surgeons that still have that or I′m not sure if I should use you because you′re a nurse. And I think there′s some surgeons that actually prefer it because they know that nurses know how operating theatres work.’ (Participant 5)
		‘We got better, and we became more useful that they kind of became more accepting.’ (Participant 1)
		‘Some would not put me down personally but make me feel as though I was taking their job.’ (Participant 2)
		‘There was lots of pushbacks from anaesthetics as well.’ (Participant 2)
		‘The people who are most negative about my practice are the ones, as they tend to be younger consultants who straddle that divide in the public and private domain with a view to being more private.’ (Participant 3)
		‘The consultant surgeons took a while to come around, but the new ones are very for it. They see the benefits to it.’ (Participant 4)
		‘The other urologists, being quite antsy about us. What if they miss it and they miss the tumour, you know, how can we trust them? You know, what′s their training? Is it going to be sufficient enough?’ (Participant 4)
	Organisational interactions	‘I think management is definitely the hardest part of it because we are employed as a nurse, but we are working for the surgeons. It′s very challenging because they don′t see what we do, they don′t know what is required for us. So, I think management, it′s definitely challenging.’ (Participant 1)
		‘We laugh, but it did take a toll on us, and it still does. We would get harassed because our theatre list was 10 minutes over, you know, the nurse unit manager would march in and say right, out everyone out. We′re done and be really aggressive and harass us and every now and then someone tries it again. Again, it could be our personality, but we feel that we need to justify ourselves and we are continually justifying ourselves and we′ll continue till the day we resign from this place.’ (Participant 4)
		‘Hospital admin was confused. You know, they didn′t really understand the role.
		Depending on how progressive the director of nursing was depended on how the hospital reacted to my application for credentialing. So, some hospitals I’m credentialed as a nurse practitioner. Some hospitals. I′m credentialed as a perioperative nurse surgeon assistant and one hospital I’m credentialed as an allied health professional because they didn′t know where to put me.’ (Participant 5)
		‘I was lucky that I was in an environment that was fully supported, the nurse unit manager, D– at L– hospital is a real motivator. She′s a, you know, a first adopter of new ideas. She′s always been that way. She′s moved on now she′s retired, but she was certainly instrumental in getting the whole thing happening at L–, the director of Gastroenterology, Dr C– M– was also used to working with advanced practice nurses in the US, so with that, she easily adopted training a nurse endoscopist.’ (Participant 3)
		‘My current nursing supervisor is very supportive. And the supervisor above that nursing supervisor is supportive. The nurse unit manager is supportive and the nurses that I work with are really lovely and supportive and they enjoy working with me.’ (Participant 3)
		‘I feel we do have the hospital support, and I certainly know that I have my direct manager support. She absolutely supports us and expects our clinical decisions. Umm. And I certainly have the head of the unit support.’ (Participant 4)
		‘The managers try and baffle us with quote unquote b– and tell us that, oh yes, we′re highly paid nurses and that you know, oh we we′re equivalent to the doctors as well. I call b– on that. There′s no way, you know, even with two Grade 5 nurses you′re paying less than a surgeon like guaranteed. So, you′re making money from us because you′re getting patients through, yet you don′t come back and give us anything back.’ (Participant 4)
		‘Little involvement from management in terms of support for my role it was I just walked into the position and that was it.’ (Participant 2)
		‘I mean, obviously they get financed to have that role within the organisation and it all depends on how passionate the directors of nursing within the organisations are towards these senior roles. Some are very passionate. Some quite obstructive. So, I think that′s probably where most of that lies.’ (Participant 2)
		‘The Australian Medical Association has been extremely obstructive and do not like the role and do not want the erosion of medical practitioners’ roles.’ (Participant 5)
		‘I think the Royal Australian College of Surgeons, acknowledges us.’ (Participant 1)
		‘The Royal College of Surgeons has been supportive to a point, so they will support nurse surgical assistance, but they won′t support nurses as primary operators.’ (Participant 5)
		‘GESA, which is the Gastroenterological Society of Australia, is very anti nurse endoscopist because their membership base is very much the private gastroenterologists, and they make a lot of money out of doing day‐to‐day diagnostics and nurses certainly threatened that practice for them. And there is a statement on the GESA website, which is very unfriendly about nurse endoscopists. It′s a position statement and I have asked them to take it down and they′ve not done that.’ (Participant 3)
		‘I didn′t find much support there [Ahpra] at all.’ (Participant 2)
		‘Ahpra, they, you know signed me off and registered me as a nurse practitioner but apart from that I don′t seek their support. Ahpra appreciating that there are those different groups of nurses that need more support.’ (Participant 3)
		‘The ANMF have been promising for a very long time to look into advanced practice nurses, that there is a deficit between those accredited to do a particular task or run a particular service like us, but who are not nurse unit managers. I have been holding hope that the ANMF will do something, but I haven′t seen anything.’ (Participant 4)
		‘I then had to get onto the Nursing and Midwifery Board. I know I wasn′t operating outside my scope of practice, but to then get clarification and have the person from the nursing and midwifery board ring the person from the Department of Veterans Affairs and tell them that I wasn′t operating outside my scope of practice.’ (Participant 5)
		‘The Nursing and Midwifery Board of Australia was good. They realised that it was within the nurses’ scope of practice to do this role, and they were supportive, but in a very passive way, so they weren′t going out there and batting for you. But if you came to them with an issue, they would help you with it.’ (Participant 4)
		‘I had issues with the Department of Veterans Affairs. They rang one of the surgeons I worked with and told him that I was operating outside of my scope of practice. The health funds and DVA have been very unsupportive and obstructive to some point.’ (Participant 5)
		‘That’s been challenging but I think they’re [accreditors] slowly starting to understand and appreciate that we are competent.’ (Participant 1)

Facilitators for nurse–surgeon integration to the Australian health system	Advocating for recognition and integration	‘It′s probably a lack of understanding about their roles.’ (Participant 3)
		‘I think more education around what the role is, a lot of people are suspicious about what they don′t understand. That was a lesson to me to actually offer lots of opportunities for training and talking to people about what that is.’ (Participant 4)
		‘I think it was inspiring to them to see that you can remain in a clinical role and advance, which is a big difference to the old way of thinking about getting along in life. As a nurse, you don′t have to be an administrator, you can be a clinician.’ (Participant 3)
		‘So, there is confusion in the healthcare system about nurse surgical assistants and nurse surgeons for the surgery like cardiac surgery.’ (Participant 5)
		‘I think nurse surgeons should be definitely doing exactly what I′m doing. I′m doing this stuff in saphenous veins and the radial arteries. And mostly because it′s not a very interesting part for them.’ (Participant 1)
		‘You′ve gotta be good. You know, only the good will survive. You know, if you′re a good surgeon, you′re gonna survive the bad ones. Kind of all get filtered out somehow. Yeah, I don′t know, just the right people have to do it, I guess.’ (Participant 1)
		‘So, I know that this is about public hospitals. In the public hospitals, the registrars and residents, they all change every three months, every six months, two years. And there′s you need the continuity of care, and you need the experience and that′s what we provide because we don′t have any desire to become a surgeon or to change specialty. You know it′s just experience. The experience that we have and benefits the team, and that′s what secures our job, I think as well.’ (Participant 1)
		‘I am aware that I have not got that medical knowledge, and I work within the parameters of my practice. However, if I had or someone had the ability, and I had approached it through a competence‐based training program. I really do believe the sky′s the limit.’ (Participant 3)
		‘The more of them that are there and practicing and doing good, the better the role would be appreciated.’ (Participant 3)
		‘Quickly being able to be performed, make a difference to the patient’s life, and remove them from the waiting lists.’ (Participant 5)
		‘I think they just kind of have to have someone to push to kind of get it and then once it′s through the door, it′s kind of done.’ (Participant 1)
		‘It′s just fighting, pushing through those barriers and pushing through those political views within the organisation.’ (Participant 2)
		‘You gotta have competencies signed off. If a surgeon wants you to do a certain thing then you have to have a structured competency. I think there definitely needs to be a credentialing pathway. Something where you have a provider number’ (Participant 3)
		‘The doctors have credentialing and pathways, and we basically had to do exactly the same training that they did. However, we have to be recredentialed every few years and there is no recredentialing body in terms of the safety standards and so forth for us.’ (Participant 2)
		‘I believe it is the suspicion that your practice is going to be substandard. And that′s why I think in training, it needs an external process where people can say oh if you were taught by this medical staff member or something. That validation that comes with expertise in your training.’ (Participant 3)
		‘There′s no overarching nationwide accreditation. Accreditation across the state. Uh Australasia. That is absolutely imperative.’ (Participant 4)
		‘I think the practice at the moment is kind of like a little bit in limbo.’ (Participant 1)
	Capacity building and professional development	‘The government and the Department of Health can assist in making these roles happen outside the big public hospitals. Then, unless that infrastructure and that financial infrastructure is in place, then there is no way that these roles can be sustainable. Nurse practitioners who are trying to work in primary healthcare clinics, you know their rebate is so low that they just can′t make a living. So, it would be the same. I operate on a number of funding models, as I said, and it′s difficult. You know, it′s difficult to make a living. I′m OK now I′m established, but setting out you really can′t rely on it as your sole income because it′s really hard to get yourself established and get enough work to actually pay the bills. So, until the government comes around to seeing that nurses can add value to the healthcare sector. Until the government can embrace the notion that nurses can work independently, be that in surgery, or be that in general practice. And until the government can override the political views of people like the AMA, then it′s not going to happen. I think that we are making inroads into getting recognition at that high government level.’ (Participant 5)
		‘More support for nurses that are practicing at such high levels because up until now I′ve been doing this well for 10 years and no one has approached me. No one has said, oh my God, what have you done? No one from AHPRA. I mean, obviously I have to submit all my documentation, but you know more interests need to be paid towards such high practicing nurses because there aren′t many of us. And you know, we′re doing a stellar job and there′s no recognition. That′s probably the word that I′m looking for. That like recognition. They don′t promote us in any way. So, there′s no promotion.’ (Participant 2)
		‘There needs to be some organisations set up that actually fights for our ability to practice.’ (Participant 2)
		‘Some collegiality and support maybe even.’ (Participant 3)
		‘We should actually have, you know, a professional organisation.’ (Participant 4)
		‘It′s gotta be down to surgeons’ support and collaboration with the nursing team.’ (Participant 1)
		‘Things are a lot smoother now. I do my job and enjoy it. I have good working relations with my colleagues, both medical and nursing.’ (Participant 2)
		‘I don′t think nurse surgeons who don′t work closely with the surgeon are going to have any joy in the next 10 years.’ (Participant 5)
		‘We have an opportunity to operate with a range of surgeons. So, I operate with six surgeons so I can give advice to them about how other people do it. You know, like you′re struggling there, oh, I′ve seen this person do it this way. I′ve seen this person do it that way and that′s really advantageous for them.’ (Participant 1)
		‘I think for me I found that the more I pitched in and helped with the changeovers and helped clean up and made it clear that I wasn′t an elitist member of the team. That I was just gonna sit over here in the corner and do nothing until it was my turn to operate. And I think you really have to demonstrate to the nurses who don′t know you how committed you are to being a member of the team.’ (Participant 5)
		‘The surgeons that I work with were amazing, so they would definitely enablers.’ (Participant 5)
		‘I would provide more support because doctors have the support, whereas I didn′t. I didn′t have support from my nursing colleagues. I was meant to have a like a mentor who was one of the divisional directors of nursing. And I met with her twice but the whole training, because she was always too busy and that not taking not saying anything bad about that about her which she was busy. I think that they could have managed that part of it a lot better.’ (Participant 2)
		‘I think having an office of the Chief Nurse role that has a place at parliamentary or within federal politics is very important. Having a nurse at the table is always very important. And then within, you know, take it down to the micro of within your own hospital facility, I think having nurse practitioners attend general meetings that look at the way strategic development of a hospital is going is very important, right down to within a department and nurses should be at those meetings so that they′re having a say about nurses in strategic development to roles that they could potentially fulfill.’ (Participant 3)
		‘If the person on the top doesn′t support, no one’s gonna support you.’ (Participant 2)
		‘Set it up with executive support right from the beginning rather than a ground up approach. They sought the support from the executive and had them give direction downwards, which I thought was a really valuable thing. I have seen it fall apart when it hasn′t come from the executive level of the hospital, nursing and medical. I think it really requires that level of commitment from higher up. I would say that executive really needs to support it and it needs to be a top‐down approach so that those nurses are more supported in those roles, particularly when they′re doing something a little bit different from what is normally within the scope of a nurse or even a nurse practitioner. So, I think it really does need executive to sort of say this is what we′re doing, and we support it fully.’ (Participant 3)
		‘Practice roles have got to come from the top down.’ (Participant 5)
		‘It was just basically talking, and I suppose building the confidence within myself to be able to do something like that.’ (Participant 2)
		‘You have to become unbelievably good at communication and convincing the nurses that you are there to add to their experience and the patients experience. I think it′s how you use your communication skills to bring that around to a positive situation than a negative situation.’ (Participant 5)
		‘I think you know you have to be open to always learning and even now 12 years down the track, we see things in the bladder we′ve never seen. So we bring the consultant up. What do we do? You know? So, we have a very open relationship and an ongoing learning relationship with the consultants, and I think that′s really important.’ (Participant 4)
		‘We don′t get any conference support. We don′t get that. That would be another thing. You know if you know you want us to present, you want us to do research, yet you don′t provide us with any resources you know we can′t even get a database going.’ (Participant 4)
		‘I guess that I had an advantage that I had 25 years of cardiac surgery behind me before I started doing it myself. I think that it would be a different experience for a nurse who hadn′t worked in cardiac surgery for an extended period of time. Because I had seen all the things that could go wrong, and I′d seen the way the surgeons, they had them go wrong, had dealt with those problems. So, I guess I had a real depth of knowledge for problem solving before I started having to do it myself.’ (Participant 5)
		‘I think a public hospital really is the best place to be training. And better, more opportunities.’ (Participant 1)
		‘I think it was adequate. I think it was very detailed. As I said, we had to have exactly the same outcomes as my medical trainees before we could practice independently.’ (Participant 2)
		‘It was adequate. I really enjoyed the training. I had a very supportive environment, and I had. Opportunities like I was treated as a trainee endoscopist. I went to meetings. I went to information sessions. I went to education meetings that were in house for the medical trainees. I was brought along and encouraged. So, I felt that was really good.’ (Participant 3)
		‘But what you gotta remember is the training that medical students get for surgery isn′t to set them up as a surgeon. It′s to give them a taste and some very basic skills. So, the training for the, you know, invasive role of harvesting conduit is really more in line with a surgical fellowship. That level of training where you′re actually training to a position where you can do it independently. So, I guess, and a lot of fellowship training is on the job training. So, it′s actually training that you do in the hospital on patients.’ (Participant 5)
		‘I think I had all stars aligned and it would been the perfect start. I didn′t have any cardiac experience at the time, but I was able to secure the job. So, I think luck was most of that.’ (Participant 1)
		‘I was lucky that I was in an environment that was fully supported. I was lucky, one of the surgeons was my trainer and he was the director of surgery.’ (Participant 3)
		‘And I think it′s also down to, you know who you are as a person, whether you can be strong. So, it′s about being the right personality for the job. You′ve gotta be strong. You′ve gotta be driven. You′ve gotta go out of your way to find things.’ (Participant 1)
		‘I think that it depends on the person who is doing the role.’ (Participant 5)

Barriers to nurse‐surgeon integration to the Australian public health system	No subtheme	‘I cannot move my service elsewhere. I′m accredited internally and that′s the only place I′m accredited.’ (Participant 4)
		‘I have to call on the nurses because they′re the ones that approve any kind of leave, but then it′s the surgical side of things that actually require not the approval, but they, they′re the ones that are kind of making the shots. So, I have to call my cardiac boss to call the nursing boss to then approve something for me. So, it′s just a massive triangle and it would be a lot easier if we were actually employed under the surgical side of things.’ (Participant 1)
		‘I feel that they are a medical association, and they have no role in stating what a nurse can and cannot do.’ (Participant 3)
		‘I think the Australian Medical Association is becoming having less of a stranglehold on what happens in the Department of Health, but they are politically very strong. So, I was recently at a cardiac conference, and they were talking about workforce planning, and you know how all these junior registrars are and senior registrars and do we need more cardiac surgeons? And what′s gonna happen at these people? Are they just going to be senior registrars forever and the person that was chairing the conference actually said, what about advanced practice nurses like, you know, they could do the job of the senior registrar both in the wards and in the operating theatre. And that was completely shut down. It was interesting that it just that wasn′t even going to be discussed in that open forum. So yeah. It is. It′s always extremely disappointing, I thought.’ (Participant 5)
		‘But there is also issues with you know payment, if I was to go public, MBS item numbers, all that kind of stuff is not accessible.’ (Participant 1)
		‘I think there are a lot of kind of financial issues with that. There′s a lot of insurance I′m sure involved so financially as well, how much do you pay them?’ (Participant 1)
		‘Because as a nurse practitioner I can do exactly the same thing in the public. I can do colonoscopy in public, but I don′t have access to item numbers to do it in private. So, it′s just the disparity is really quite substantial.’ (Participant 2)
		‘There definitely needs to be more money and particularly when the original idea behind at least the nurses cystoscopist was to free up the doctor′s time to actually go into main theatres to actually do you know, more, more training for within because they weren′t doing enough training, enough operating time because there was thousands of flexis that they had to do.’ (Participant 4)
		‘I don′t think there′s gonna be enough money in the world to keep them within nursing. Because you know, a lot of the networks really haven′t been treating us as well as they could have been. And. Yeah, so I think. COVID has sullied the waters in a lot of ways and people just aren′t interested anymore. In focusing on nursing as a career and advancing their practice.’ (Participant 4)
		‘I think once we get a rebate for the patients, it will make a huge difference.’ (Participant 5)
		‘The legislation has to be in place to support the financial ability of nurses to work in the role.’ (Participant 5)
		‘And then, of course, there was the tall poppy syndrome from my nursing colleagues as well where they would, if there was any questions, they would bypass me and go to the doctor that was working next door. Until I said guys, you need to come to me. If there′s anything that you need to know. Any grievances, anything that′s going on, you need to ask me because I′m the one that′s in charge here and I′m responsible in the end.’ (Participant 2)
		‘I do think it′s a tall poppy syndrome that does exist within nursing.’(Participant 4)
		‘There will always be an element of tall poppy syndrome as an Australian cultural thing and if you are trying to do something new and broaden the scope of practice of nurses, there are always going to be nurses that will cut you down.’ (Participant 5)
		‘They [other consultants] can′t get their heads around that and there is somewhat a superiority complex in regard to that.’ (Participant 4)
		‘I don′t think you′d need to watch as many like I don′t think you′d need to observe as many [surgeries].’ (Participant 4)
		‘I think that size of the institution, the healthcare institution and the. The other properties of the healthcare institution. So, if you′re talking a major public training hospital, then yeah, there′s gonna be a lot of processes that you have to work through which could be seen as obstructive. To getting a role like this off the ground. If you′re talking a small private. Umm, facility that′s owned by a couple of surgeons then. Yeah, they′re gonna want to get this off the ground because they can see that it′s gonna benefit them. So I think you know, you need to look at what area of the healthcare sector you′re looking at and. In what particular circumstance you′re trying to bring the role in, yeah.’ (Participant 5)

### 3.1. Theme 1: Nurse‐Surgeon Interactions

This theme explores how nurse‐surgeons interacted with other health professionals, divided into two subthemes. The first subtheme examines nurse‐surgeon interactions within the surgical team, while the second focuses on engagements with broader healthcare organisations.

#### 3.1.1. Subtheme 1: Interactions With Healthcare Professionals

Interactions within the surgical team were influenced by colleagues’ professional backgrounds. Participant 1 reflected, ‘*There was definitely a head-to-head with the nurses… quite challenging… I was almost stepping on their toes… It was quite stressful… threatening for them*.’ Participant 3 noted support from a colorectal surgeon: ‘*It was entirely collaborative… I was really supported in that environment.*’ Participants also faced challenges like ‘tall poppy syndrome’, where Participant 2 mentioned, ‘*Until I said, guys, you need to come to me… I’m the one that’s in charge here.*’

The context of these interactions, whether during training or independent practice, also affected experiences. Participant 5 remarked, ‘*The surgeons that I knew well… probably didn’t change over… my experience.*’ Participant 5 shared, ‘*They were a bit reserved… but gave me the leeway… they often say, thanks for your help.*’

Familiarity with colleagues impacted interactions as well. Participant 4 stated, ‘*They [junior physicians] don’t understand what we do… There’s a superiority complex regarding that.*’ This led to initial role confusion, highlighted by Participant 2, who said, ‘*It’s not something that we’re taught… giving a diagnosis… that’s not something that we’re trained to do.*’

As participants acclimated to their roles, confusion diminished, leading to greater recognition of nurse‐surgeons’ value. Participant 1 noted, ‘*And now the nurses are grateful for… communication’.* Participant 2 stated, ‘*I think they now see me as that role model within the nursing profession.*’ Participant 3 observed, ‘*A lot of the nurses were inspired… I think half our unit went and did… postgraduate training*.’

#### 3.1.2. Subtheme 2: Organisational Interactions

Participants faced challenges in their interactions with administrators due to a lack of understanding of the nurse‐surgeon role. Experiences were influenced by management figures who decided whether this role would be implemented. Participant 5 stated, ‘*Hospital admin was confused… they didn’t really understand the role. Depending on how progressive the director of nursing was… some hospitals I’m credentialed as a nurse practitioner… others as a perioperative nurse surgeon assistant… one as an allied health professional because they didn’t know where to put me.’* This inconsistency highlighted the impact of administrative attitudes, with Participant 2 noting, ‘*…it all depends on how passionate the directors of nursing… are towards these senior roles. Some are very passionate… some quite obstructive…*’

Interactions with external organisations complicated the landscape for nurse‐surgeons. Participants viewed medical associations as antagonistic, often resisting changes that could threaten traditional medical roles. Participant 5 remarked, ‘*The Australian Medical Association has been extremely obstructive… do not want the erosion of medical practitioners’ roles’.* In contrast, nursing organisations offered passive support or indifference. Participant 4 reflected, ‘*The Nursing and Midwifery Board of Australia… realised that it was within the nurses’ scope of practice… supportive, but… not going out there and batting for you…*’ This dichotomy illustrates the complexities nurse‐surgeons faced in seeking recognition and support within the healthcare system.

### 3.2. Theme 2: Facilitators for Nurse‐Surgeon Integration Into the Australian Health System

This theme highlights the factors facilitating the integration of nurse‐surgeons into the Australian health system, divided into two subthemes. The first, advocating for recognition and integration, emphasises proactive measures to legitimise the role. The second, capacity building and professional development, focuses on equipping nurse‐surgeons with the skills and support needed to succeed. Together, these subthemes reflect a comprehensive approach to integrating nurse‐surgeons into the healthcare system.

#### 3.2.1. Subtheme 1: Advocating for Recognition and Integration

This subtheme highlights efforts to raise awareness of the nurse‐surgeon role, establish a standardised national credentialing pathway, engage with government bodies and create a dedicated organisation for nurse‐surgeons. Participants emphasised the importance of education in alleviating suspicion about the role. Participant 4 stated, ‘*I think more education around what the role is… a lot of people are suspicious about what they don’t understand.*’ Effective communication is also crucial, as noted by Participant 5, ‘*You have to become unbelievably good at communication… to bring that around to a positive situation.*’

Participants stressed the need for standardised national credentialing as a mean to validate the training and practice of nurse‐surgeons. A consistent accreditation process is vital for maintaining high standards and fostering trust. Participant 4 highlighted, ‘*There’s no overarching nationwide accreditation… that is absolutely imperative.*’ This external validation process helps reassure others regarding the quality of nurse‐surgeons’ training.

Participants also pointed out the necessity of government involvement and the establishment of a dedicated professional organisation for nurse‐surgeons. Government support is seen as essential for the role’s sustainability, particularly outside large public hospitals. Participant 2 remarked, ‘*There needs to be some organisations set up that actually fights for our ability to practice.*’

Recognition at the government level is crucial to overcoming political opposition from organisations like the Australian Medical Association. Participant 5 noted, ‘*Until the government sees that nurses can add value… it’s not going to happen… I think we are making inroads into getting recognition at that high government level.*’ This recognition is fundamental for securing the nurse‐surgeon’s place within the healthcare sector.

#### 3.2.2. Subtheme 2: Capacity Building and Professional Development

This subtheme focuses on collaboration to build the nurse‐surgeon workforce and develop the profession. Key elements include nursing representation in high‐level health discussions, effective communication, ongoing learning, acquiring relevant clinical experience and facilitating on‐the‐job training. Participant 3 emphasised the need for nursing representation, stating, ‘*I think having an office of the Chief Nurse role… having a nurse at the table is always very important… nurses should be at those meetings so that they’re having a say about nurses in strategic development.*’

Executive support is essential for successful nurse‐surgeon roles. Participant 5 remarked, ‘*Practice roles have got to come from the top down,’* while Participant 3 noted, *‘Set it up with executive support right from the beginning… I have seen it fall apart when it hasn’t come from the executive level*.’ This support ensures that nurse‐surgeons receive the recognition and backing they need to thrive in their roles.

Effective communication is crucial for fostering a positive atmosphere within the surgical team. Participant 5 highlighted, ‘*You have to become unbelievably good at communication… to bring that around to a positive situation.*’ Additionally, continuous learning and maintaining open relationships with consultants are vital for nurse‐surgeons’ development, as stated by Participant 3: ‘*I had a very supportive environment… I was brought along and encouraged.*’

Relevant clinical experience is also critical for independent practice. Participant 5 shared, ‘*I had an advantage that I had 25 years of cardiac surgery behind me before I started doing it myself*.’ On‐the‐job training mirrors surgical fellowship models, emphasising practical experience. Participant 2 noted, ‘*As I said, we had to have exactly the same outcomes as my medical trainees before we could practice independently*’, while Participant 5 added, ‘*A lot of fellowship training is on-the-job training… training that you do in the hospital on patients.*’

### 3.3. Theme 3: Barriers to Nurse‐Surgeon Integration in the Australian Public Health System

This theme highlights the barriers nurse‐surgeons face in the Australian healthcare system. Participants identified several challenges, including geographical limitations that restrict service locations. Participant 4 stated, ‘*I cannot move my service elsewhere. I’m accredited internally and that’s the only place I’m accredited*.’

Political obstruction from medical associations also poses a significant hurdle. Participant 3 noted, ‘*I feel that they [Australian Medical Association] are a medical association, and they have no role in stating what a nurse can and cannot do*.’ Additionally, regulatory challenges create disparities between public and private practices. Participant 2 explained, ‘*As a nurse practitioner I can do exactly the same thing in the public… but I don’t have access to item numbers to do it in private. So, it’s just the disparity is really quite substantial.*’

Finally, a superiority complex among physicians hampers the collaboration and recognition of nurse‐surgeon expertise. Participant 4 remarked, ‘*They [other consultants] can’t get their heads around that [nurses performing surgeries], and there is somewhat a superiority complex in regard to that.*’ This reflects the need for attitudinal changes within the medical community to facilitate integration.

## 4. Discussion

This study examined the experiences and perceptions of nurse‐surgeons in the Australian public health system, highlighting initial resistance from nurses, junior physicians and other consultants due to concerns about competence, role conflicts and resistance to change [26]. The introduction of nurse‐surgeon roles challenged established norms and created uncertainty among surgical staff. Concerns about nurse‐surgeons’ surgical capabilities, despite their specialised training, also contributed to this apprehension. However, as attitudes shifted over time, addressing these concerns and fostering open communication proved essential for the successful integration of nurse‐surgeons within the healthcare system [27].

Initially, nurse‐surgeons faced resistance from nurses, junior physicians and consultants. However, participants reported strong support from their physician supervisors, which can be attributed to several factors. First, supervisors value mentoring nurse‐surgeons, contributing to the development of skilled professionals and safer patient outcomes. Second, this support helps expand the surgical workforce, improving access to care. Third, nurse‐surgeons are cost‐effective, performing surgeries at lower salaries than specialised surgeons. Finally, their involvement allows specialised surgeons to focus on complex cases, improving healthcare efficiency and timely access to surgeries [28, 29].

Participants noted that prior exposure to nurse‐surgeons positively influenced support from physician–supervisors and other surgical team members. Direct experience with nurse‐surgeons’ skills and professionalism, gained through collaboration, enhances understanding and appreciation of their contributions. Successful teamwork between surgeons and nurse‐surgeons builds trust in a team‐based approach to patient care. This firsthand experience encourages advocacy for nurse‐surgeons among physicians and healthcare team members, highlighting their role in achieving optimal surgical outcomes [30].

The integration of nurse‐surgeons into the Australian healthcare system depends on several factors, including awareness of the role, recognition of its value, advocacy, collaboration, a standardised national credentialing pathway, on‐the‐job training, relevant clinical experience, continuous learning, effective communication, nursing representation, a top–down approach, a dedicated organisation for nurse‐surgeons and government involvement. These factors are essential for improving patient outcomes by creating a framework that supports the effective inclusion of nurse‐surgeons in surgical healthcare [31, 32].

Raising awareness of nurse‐surgeon roles is essential and can be achieved through educational programs, collaborative campaigns, media engagement, sharing success stories, addressing concerns and advocating for a supportive environment [33]. Implementing a standardised national credentialing pathway for nurse‐surgeons would establish consistent criteria for education and training, promote workforce mobility and patient safety and facilitate integration and career advancement [31]. A top–down approach led by senior leadership can help navigate challenges, allocate resources and align with organisational goals [32]. Creating a dedicated organisation for nurse‐surgeons would provide a platform for collaboration, professional growth, training and research to enhance surgical practice [36]. Additionally, involving nurses in the strategic development of nursing practices and collaborating with the Australian government would strengthen the healthcare workforce and improve the overall healthcare system [37].

The integration of nurse‐surgeons into the Australian healthcare system faces several barriers. Geographical limitations restrict the distribution of surgical services and career mobility, complicating workforce planning [36]. Nurse‐surgeons face challenges similar to those of nurse practitioners, particularly in rural areas, where regulatory restrictions limit their scope of practice [37]. Both roles confront barriers that hinder workforce mobility and access to essential healthcare services. Addressing these challenges is crucial for effectively utilising advanced nursing roles in the healthcare system. Developing a national credentialing pathway could help alleviate these issues by standardising training and addressing workforce shortages.

Political opposition from influential medical associations hinders the recognition of advanced nursing roles [38]. Collaboration between nurses and physicians is vital for addressing workforce shortages and improving patient access to surgical services. This requires open dialogue and evidence‐based advocacy to effectively utilise nurse‐surgeons’ skills. Resistance to change can also impede the acceptance of nurse‐surgeons [39]. Overcoming this requires a cultural shift that fosters professional growth and innovation. Additionally, some surgeons’ superiority complex can create barriers to collaboration and teamwork [45]. Promoting mutual respect and acknowledging the contributions of all healthcare team members is essential for breaking down these barriers.

The role of nurse‐surgeons holds compelling global relevance, particularly in addressing surgical workforce shortages and improving access to essential care in diverse healthcare contexts. Findings from this study highlight barriers, such as geographical maldistribution of health services, regulatory restrictions and resistance from professional bodies, which mirror challenges faced by advanced practice nurses internationally where similar inequities persist [38, 46]. In low‐ and middle‐income countries, where surgical capacity is critically limited, adopting nurse‐surgeons could provide a sustainable workforce solution to reduce treatment delays and enhance equity of access [42–44]. In high‐income settings, nurse‐surgeons could strengthen service delivery in underserved areas, while fostering collaborative practice models that bridge gaps between nursing and medicine [45, 46]. Standardising education and credentialing pathways across borders would not only enhance role legitimacy but also facilitate workforce mobility, knowledge exchange and global health system resilience. Therefore, positioning nurse‐surgeons as an internationally recognised advanced practice role is a necessary step to future‐proof surgical care delivery and respond to the growing global demand for accessible, safe and timely surgery.

This study demonstrates strengths such as credibility, transferability, dependability and confirmability. The detailed contextual information on nurse‐surgeon experiences enhances transferability, enabling readers to assess relevance in similar contexts. Consistency in data collection and analysis ensures dependability, fostering confidence in the findings’ reliability. Confirmability is achieved through transparent data interpretation, contributing to the robustness of the study’s outcomes. However, several limitations also affected the results. Potential bias from researcher‐specific factors, such as demeanour and interviewee perceptions, could lead to incomplete or skewed data, impacting the findings’ accuracy and validity. Additionally, the focus on individual experiences may overlook broader systemic issues affecting nurse‐surgeons. The limited number of participants (five) reflects the small population of nurse‐surgeons in Australia, which, combined with the participants’ varying abilities to articulate their experiences, may further affect the richness and robustness of the data collected. Together, these strengths and limitations shape the overall trustworthiness and applicability of the study’s findings.

### 4.1. Recommendations for Further Research

This qualitative study highlights several recommendations for future research. First, examining the experiences of nurse‐surgeons across various regions and healthcare settings would provide a more complete understanding of the challenges and successes they encounter, helping to identify specific barriers and facilitators. Second, conducting longitudinal studies to track nurse‐surgeons’ career paths could reveal the long‐term impact of their roles on professional growth, job satisfaction and patient care. Lastly, exploring the perceptions of other healthcare professionals about nurse‐surgeons would offer insights into their acceptance and integration within surgical teams. Understanding these perspectives could help address barriers and foster collaboration, enhancing the implementation of nurse‐surgeons in the Australian public health system and beyond.

### 4.2. Implications for Policy and Practice

Identifying the facilitators and barriers faced by nurse‐surgeons can inform targeted policies to support their integration into the healthcare workforce and address systemic issues. Insights from this study can guide policymakers in promoting collaboration between nurse‐surgeons and other professionals, as well as highlight the need for a national credentialing pathway and tailored continuing education programs. Recognising nurse‐surgeons in healthcare organisations and at executive levels can also foster a culture that values their expertise. Additionally, the study reveals areas for professional growth, guiding the development of support programs and mentorship opportunities. Understanding factors that enhance job satisfaction—such as collaboration, value recognition and advocacy—can help improve retention rates and create a resilient surgical workforce, benefiting all healthcare practitioners in building effective teamwork.

## 5. Conclusion

This study highlights significant policy implications for the integration of nurse‐surgeons into the Australian healthcare system. By identifying key facilitators and barriers, the research informs public policy aimed at enhancing collaboration between nurse‐surgeons and other healthcare professionals. Developing a national credentialing pathway is essential for standardising training and promoting workforce mobility, ultimately improving patient access to surgical care. Social policy should prioritise recognition and representation of nurse‐surgeons within healthcare organisations, fostering a culture that values their contributions. Organisational policies must emphasise mentorship and professional development programs to support nurse‐surgeons and improve job satisfaction, retention and workforce sustainability. Management models and frameworks can benefit from integrating nurse‐surgeons into strategic discussions, ensuring their skills are utilised effectively. Additionally, education models should focus on tailored continuing education programs for nurse‐surgeons, aligning with accreditation standards that reflect their unique role in healthcare. Overall, these policy recommendations can significantly enhance the effectiveness of nurse‐surgeons, contributing to improved surgical outcomes and access to care across diverse settings.

## Conflicts of Interest

The authors declare no conflicts of interest.

## Funding

The first author (TG) received funding from the Australian Government Research Training Program to complete this research.

## Supporting Information

Additional supporting information can be found online in the Supporting Information section.

## Supporting information


**Supporting Information 1** Supporting Information 1 COREQ.docx. A copy of the completed consolidated criteria for reporting qualitative research checklist.

**Supporting Information 1** Supporting Information 2 IG.docx. A copy of the interview guide.

**Supporting Information 1** Supporting Information 3 RS.docx. A copy of TG’s reflexivity statement.

## Data Availability

The data that support the findings of this study are available upon request from the corresponding author. The data are not publicly available due to privacy or ethical restrictions.
