# Supplementary material for: Nurse‐Surgeons’ Experiences Working in the Australian Public Health System: A Qualitative Exploration
Source: J Nurs Manag. 2026 Jan 16;2026:2341474. doi: 10.1155/jonm/2341474 (PMC12811622; doi:10.1155/jonm/2341474)
Supplement: Supplementary file 2 — Supporting Information 1 Supporting Information 2 IG.docx. A copy of the interview guide. [file JONM-2026-2341474-s002.docx]

Supplementary material 2. Interview guide

Interview protocol

**Research study title: Nurse-surgeons in the Australian public health system Phase 2 (qualitative phase): Semi-structured interview**

**Section 1. Basic information**

| Date of the interview: |  |
| --- | --- |
| Time of the interview: |  |
| Location of the interview: |  |
| Name of the interviewer: |  |
| Name of the interviewee: |  |
| Actual length of the interview: |  |
| File name of the audio-recording: |  |
| File name of the transcription: |  |

**Section 2. Introduction**

Interviewer checklist:

1. Introduce yourself
2. Discuss the purpose of the study and the structure of the interview
   1. *To explore the experiences of nurse-surgeons in the Australian public health system*
   2. *The interview will take approximately one hour to complete*
3. Show the interviewee’s signed informed consent
4. Explain the following terminologies: Surgery, nurse-surgeon
5. Ask the interviewee for any questions before starting

*Interviewee question/s:*

# Section 3. Opening question

Tell me about yourself (i.e., job, what the job entails, what your usual day at work looks like)

# Section 4. Content questions

This will emerge after completion of Phase 1

1. How did you become a nurse-surgeon?
2. Can you describe the beginning of your career as a nurse-surgeon? What were the enablers, drivers, motivators, disablers, etc.
3. What were your memorable experiences being a nurse doing a physician’s role
4. How did you work out your training and education? Who were the key players in the decision-making stage of the training programme?
5. What do you think about the training you received in terms of preparation for the role?
6. If you were to redo your nurse-surgeon training, what would you do differently?
7. How did your nursing colleagues view your role at the beginning of your career? Was there a change over the years?
8. How did the surgeons view your role at the beginning of your career? Was there a change over the years?
9. How did the management view your role at the beginning of your career? Was there a change over the years?
10. We generally received survey responses indicating good to excellent support from **nurses** but there are some as well who responded that they received poor or terrible from their organisations, what do you think are the cause of this?
11. We generally received survey responses indicating good to excellent support from **management** but there are some as well who responded that they received poor or terrible from their organisations, what do you think are the cause of this?
12. We generally received survey responses indicating good to excellent support from **surgeons** but there are some as well who responded that they received poor or terrible from their organisations, what do you think are the cause of this?
13. Can you describe the support you receive from nursing unions? Ahpra? Accreditors? Medical societies and organisations?
14. Survey responses indicate that there are many limitations in the scope of practice of nurse-surgeons due to the role being not widely recognised. What do you think needs to be done for nurse-surgeons to be recognised fully in Australia?
15. Politics was provided by survey respondents as one of the main drivers of the role’s acceptance in the organisation, what do you think of this statement?
16. What do you think are the surgeries that nurse-surgeons should be trained to perform within your specialty and why do you think so?
17. In other countries, nurse-surgeons perform surgeries such as caesarean sections, hysterectomies, laparotomies, hernia repair, appendicectomies. Do you think in the future, this will be implemented in Australia as well?
18. What could be done to ensure the security of your role within your organisation, and in the Australian public health system?
19. What do you think is the future of nurse-surgeons in Australia?
20. What do you think needs to be done to futureproof the roles and practice of nurse- surgeons in Australia?

# Section 5. Probing questions

A reminder to ask for more information or further explanation of ideas

*Tell me more…*

*I need more detail…*

*Could you explain your response more? What does that mean?*

# Section 6. Closing instructions

Interviewer checklist

1. Thank you for your time.
2. Assure the interviewee of the confidentiality of the interview
3. Explain that the results will be publicly available once the study has been published. Assure the interviewees that the publication will not identify them in any way.
4. Offer to send the abstract of the final study
5. Explain what happens next.
6. Answer any questions.

# Section 7. Researcher observations

**Descriptive notes** (*for example, description of the background, comments about sounds that were not created by the interviewee, and accounts of any specific events or activities during the interview*)

**Reflexive notes** (*for example, author’s personal feelings, ideas, hunches and prejudices during the interview*)
