# Supplementary material for: Nurse‐Surgeons’ Experiences Working in the Australian Public Health System: A Qualitative Exploration
Source: J Nurs Manag. 2026 Jan 16;2026:2341474. doi: 10.1155/jonm/2341474 (PMC12811622; doi:10.1155/jonm/2341474)
Supplement: Supplementary file 3 — Supporting Information 1 Supporting Information 3 RS.docx. A copy of TG’s reflexivity statement. [file JONM-2026-2341474-s003.docx]

## **Supplementary material 3. Reflexive statement of the author**

“*I am a Filipino cisgendered male Registered Nurse with 14 years’ combined experience in perioperative nursing, perioperative service management and research. I have worked as a Theatre Registered Nurse (Philippines, New Zealand, Australia), Theatre Nurse-in-Charge (New Zealand), Perioperative Nurse Educator (Australia), Theatre Floor Coordinator (Australia), Nurse Unit Manager – Operating Theatres (Australia), Perioperative Service Manager (New Zealand), and Director of Nursing (Australia). My credentials are Bachelor of Science in Nursing completed in the Philippines, Postgraduate Diploma in Health Sciences (major in Advanced Nursing) completed with merit in New Zealand, Master of Management (Health Service Management) completed with distinction in New Zealand, and an ongoing Doctor of Philosophy currently being completed in Australia. At the time of the interviews, my occupation was part-time Community Manager – Asia Pacific Region for a non-profit organisation that focuses on scientific evidence synthesis. I did not establish a professional relationship with any of the participants prior to the study commencement. However, I introduced myself to the participants via the participant information letter and at the beginning of each interview. The participants were made aware of my experience, credentials, as well as the reasons for conducting the research as a requirement to complete my Doctor of Philosophy study. As an experienced perioperative nurse, I have a good grasp of the material and human resources needed for optimal perioperative service delivery. However, considering that I was trained and had initial clinical experience overseas, I may be biased in perceiving the differences in clinical practise between Australia and overseas. As English is my second language and I did not grow up in Australia, I assumed that I may have some difficulty in understanding the subtleties, nuances and cultural references in the Australian English language.*
